# BS-Seeker3: ultrafast pipeline for bisulfite sequencing

**DOI:** 10.1186/s12859-018-2120-7

**Published:** 2018-04-03

**Authors:** Kevin Yu Yuan Huang, Yan-Jiun Huang, Pao-Yang Chen

**Affiliations:** 1 Institute of Plant and Microbial Biology, Academia Sinica, Taipei, Taiwan; 2 Department of Biomedical Engineering, Johns Hopkins University, Baltimore, MD USA

## Abstract

**Background:**

DNA methylation is an important epigenetic modification critical in regulation and transgenerational inheritance. The methylation level can be estimated at single-nucleotide resolution by whole-genome bisulfite sequencing (BS-seq; WGBS). Current bisulfite aligners provide pipelines for processing the reads by WGBS; however, few are able to analyze the BS-seqs in a reasonable timeframe that meets the needs of the rapid expansion of epigenome sequencing in biomedical research.

**Results:**

We introduce BS-Seeker3, an extensively improved and optimized implementation of BS-Seeker2 that leverages the available computational power of a standard bioinformatics lab. BS-Seeker3 adopts all alignment features of BS-Seeker2. It performs ultrafast alignments and achieves both high accuracy and high mappability, more than twice that of the other aligners that we evaluated. Moreover, BS Seeker 3 is well linked with downstream analyzer MethGo for up to 9 types of genomic and epigenomic analyses.

**Conclusions:**

BS-Seeker3 is an accurate, versatile, ultra-fast pipeline for processing bisulfite-converted reads. It also helps the user better visualize the methylation data.

**Electronic supplementary material:**

The online version of this article (10.1186/s12859-018-2120-7) contains supplementary material, which is available to authorized users.

## Background

DNA methylation is an important epigenetic control that plays a major role in gene expression, splicing, and genomic imprinting. Current bisulfite conversion, coupled with next-generation sequencing (NGS)-based methods, e.g., whole-genome bisulfite sequencing (WGBS) and reduced representation bisulfite sequencing (RRBS), are able to profile genome-wide DNA methylation at single base-pair resolution. Subsequent analysis of NGS data proceeds with the alignment of the bisulfite reads. However, sodium bisulfite treatment converts each unmethylated cytosine (C) to uracil, so an aligner needs to allow a thymine (T) in the read to match to a C in the reference genome when an unmethylated C occurs.

Since the early 2010s, various algorithms have been proposed to accomplish such alignments; among them, BS-Seeker, Bismark, and BSMAP were the earliest developed and are the most commonly used [1–3]. The former two employ an *“in silico* bisulfite conversion” strategy that results in a three-letter genome, where all the Cs in both the reads and the reference are converted to Ts prior to alignment. In contrast, BSMAP aligns with a wildcard approach. The recent version of BS-Seeker, BS-Seeker2, is arguably one of the most versatile bisulfite aligners [4]; it can map reads from both WGBS and RRBS, allows gapped local alignment, and supports a suite of traditional DNA aligners. Additionally, the advances in high-throughput sequencing technologies in recent years had significantly lowered sequencing cost and affordability. Current bisulfite aligners need to be updated to process this out-burst of information in a timely manner.

Here, we introduce BS-Seeker3, an extensively improved and optimized implementation of BS-Seeker2 that leverages the available computational power of a standard bioinformatics lab. BS-Seeker3 adopts all alignment features of BS-Seeker2, some of them include the support for local and gapped alignment, RRBS mapping, and built-in adapter trimming [4].

BS-Seeker3 also incorporates a series of new features to achieve significantly faster speed and better accuracy compared to other available bisulfite aligners. It is 1.5X faster than BSMAP, 10X faster than Bismark and Brat-nova, and maps twice as many reads as either of those aligners [2, 3, 5]. BS-Seeker3 also offers downstream analysis of bisulfite read data to further investigate bisulfite conversion efficiency and to visualize the methylation pattern after alignment. It is also well integrated with downstream methylation analyzer MethGo to provide a variety of genomic and epigenomic analyses [6].

## Implementations

### Improved indexing/high-throughput reference genome processing

**Fig. 1:**
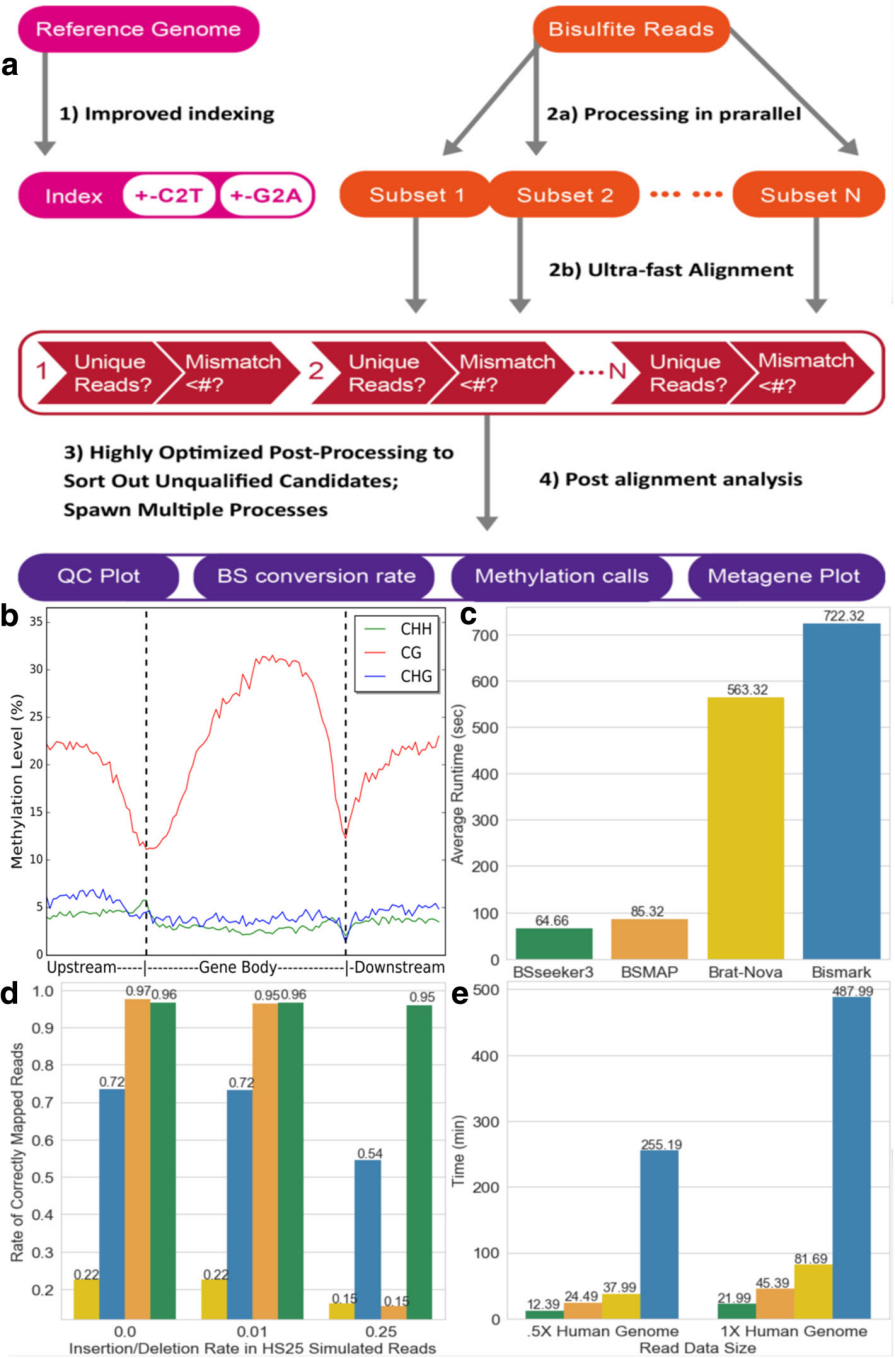
Summary of BS-Seeker3 pipeline and performance. **a** Schematic flow chart of BS Seeker 3 with improved indexing, data processing, fast alignment and post-alignment analyses (**b**) Metaplot of Methylation level: This metaplot presents the average methylation level distribution within a user-specified genomic structure (e.g., coding genes) in *Arabiodopsis thalania*. CG denotes a CpG dinucleotide, CHG denotes a cytosine next to a H where H stands for A, C, or T.and then a guanine, CHH denotes a cytosine next to two H bases (**c**) Average user runtime of the four aligners on 10 M simulated HiSeq 2500 Arabidopsis reads. **d** Percentage of the 10 M simulated HiSeq2500 reads that were mapped correctly across various reads complexity level. **e** Average runtime of four aligners on directional BS-seq reads from real human data [3]

To improve the efficiency of processing alignments, BS-Seeker3 concatenates the Watson and Crick strand sequences and builds a single index, instead of two separated indexes, as are generally used (Fig. 1a) [7]. The index is built based on the C-to-T converted sequence from each strand direction. Therefore, one read needs only one alignment, conserving 44% of raw-reads mapping time and 7% of overall runtime (Additional file 1: Fig. S1 and Method S1).

### Ultrafast alignment and local alignment through Ukkonen algorithm

BS-Seeker2 had coupled with major conventional aligners including SOAP, Bowtie, and Bowtie2 to perform 3-letter alignment. BS-Seeker3 incorporates SNAP, which employs a hash-based index of short substrings of the reference genome [8]. During alignment, SNAP breaks a read into substrings and queries their locations. Unlike other hash-based aligners, SNAP encodes substrings of greater length, significantly reducing the number of false-positive hits. BS Seeker and BS Seeker2 utilize a Smith-Waterman approach to conduct local alignment for a candidate match, which is on the order of quadratic time complexity. BS Seeker3 now checks for local edit distance using the Ukkonen Algorithm, which is in the realm of linear time complexity and thus significantly more efficient than the previous strategy [9].

### A heavily optimized pipeline

As shown in Fig. 1a the post-processing step is responsible for a large proportion of runtime. For every candidate match, the pipeline checks for the inexact match between a read C and a read T and re-calculates the mismatch number. We assumed that mismatches on an alignment are randomly distributed and evaluated each position in a random order. Furthermore, we set an upper bound on the mismatch number to reduce unnecessary calculations. We optimized this and similar bottlenecks and decreased runtime by ¼ (Additional file 1: Figure S6 and Method S1). Furthermore, as the available computational resources for a typical bioinformatics lab has grown drastically, BS-Seeker3 now divides a large read file into smaller files and leverages the high memory capacity of an average server to load multiple indexes simultaneously and process each smaller file in parallel. An user is allowed to further optimize BS-Seeker3 performance based on their computational resources. In conclusion, BS-Seeker3 is an improved version of the previous BS-Seekers, now including features from both the C and Python languages.

### Post-alignment data analysis

BS-Seeker3 offers a quality control plot based on the average rate of mismatch per read position (Additional file 1: Figure S2A) which allows the assessment of library and sequencing quality. Furthermore, BS-Seeker3 provides a unique feature to estimate bisulfite conversion efficiency, if the library contains spike in from lambda phage DNA (Additional file 1: Figure S2B). DNA of lambda phage is free of DNA methylation, so in an ideal situation all cytosine of the genome should turn into uracil. Any unconverted cytosine thus reveals the failed bisulfite conversion that may bias the methylation analysis. BS-Seeker3 also outputs a genome-wide view of methylation levels (Additional file 1: Figure S2C) and the distribution of methylation in an user-specified genomic structure such as the metagene plot (Fig. 1b), allowing timely investigation of DNA methylation at specific genomic elements. The output files from BS Seeker3 can be directly used by other downstream data analyzers such as MethGo which carries out up to 9 types of genomic and epigenomic analyses [6].

## Results

To evaluate BS-Seeker3 performance, we benchmarked its default setting against major bisulfite aligners, Bismark, BSMAP, and Brat-nova, using their default parameters (Additional file 1: Method S2) with 20 cores on an 80 cores server. The default settings were used, so the performance could be generalized to an arbitrary novel dataset where the optimal parameters would be unknown. We ran all aligners on real human reads to examine the mapping efficiency (user runtime), and on a series of synthetic HiSeq-2500 and HiSeq-1000 reads with different degrees of data complexity from the *Arabidopsis* library to compare the mapping accuracy and overall performance (Additional file 1: Method S3) [10, 11]. The different levels of complexity were simulated by varying the single base insertion/deletion rate when generating simulated reads. We recorded the percentage of reads mapped correctly, the user runtime, and the mapability (Additional file 1: Method S4).

BS-Seeker3 performed the fastest on average on synthetic read data (Fig. 1c, Additional file 1: Figure S5). Bseeker3 is 10X faster than Bismark and 9X faster than Brat-nova. Even though BSMAP’s performance parallels BSseeker3’s in both speed and accuracy (Fig. 1d) with HiSeq2500 reads at low indel rates, BSMAP mapped significantly fewer reads correctly at higher level of data complexity. When the indel rate rose to .025, BSMAP’s accuracy dropped sharply and mapped even fewer reads than Bismark or Brat-nova. On the other hand, BS-Seeker3 consistently mapped more than 90% of the reads correctly at all indel rates. In brief, BS-Seeker3 would be much more suitable to process data with high complexity where a high insertion/deletion is expected.

To showcase the feasibility of BS-Seeker3 on real-time data from a large genome, we downloaded human data sets to create two data sets of increasing sizes (.5X Human Genome and 1X Human Genome) (Fig. 1e). BS-Seeker3 achieved the fastest speed on these data, followed by BSMAP, Brat-nova and Bismark. As a matter of fact, on the human data set, BS-Seeker3 performed at least twice as fast as the other aligners, including BSMAP. Because BSMAP builds its index online, the performance gap between BS-Seeker3 and BSMAP widens as the genome size increases.

## Conclusion

In conclusion, BS-Seeker3 is an accurate, versatile, ultra-fast pipeline for processing bisulfite-converted reads. It also helps the user better visualize the methylation data.

## Additional file

Additional file 1: Supplementary Information; supplementary materials to BS-Seeker3 project. (DOCX 1032 kb)
