# Supplementary material for: BS-Seeker3: ultrafast pipeline for bisulfite sequencing
Source: BMC Bioinformatics. 2018 Apr 3;19:111. doi: 10.1186/s12859-018-2120-7 (PMC5883884; doi:10.1186/s12859-018-2120-7)
Supplement: Supplementary file 1 — Supplementary Information; supplementary materials to BS-Seeker3 project. (DOCX 1032 kb) [file 12859_2018_2120_MOESM1_ESM.docx]

Supplementary Data for:

Title: BS-Seeker3: Ultra-fast pipeline for bisulfite sequencing

**Author:** Kevin Y. Huang^1, 2^, Yan-Jiun Huang^1^, Pao-Yang Chen^1,*^

^1^Institute of Plant and Microbial Biology, Academia Sinica, Taipei, Taiwan

^2^ Department of Biomedical Engineering, Johns Hopkins University, Baltimore, MD, USA

**Contents:**

**Supplementary Method:**

**Method 1.** BS-Seeker 3 Pipeline.

**Method 2.** Command Used to Run Each Benchmark Aligner.

**Method 3.** Method used to generate synthetic *Arabiodopsis* reads.

**Method 4.** Method used to prepare real human bisulfite read datasets.

**Supplementary Figures:**

**Figure S1.** Amount of runtime conserved by Improved Indexing.

**Figure S2.** Additional analysis of BS-seq Data

**Figure S3.** Mapibility rate of the 10M simulated HiSeq1000 reads by BSseeker3, BSseeker2, BSMAP, Brat-Nova, and BSMAP across different complexity level.

**Figure S4.** Mapibility rate of the 10M simulated HiSeq2500 reads by BSseeker3, BSMAP, Brat-Nova, and BSMAP across different complexity level.

**Figure S5.** (**C**) Average user runtime of the four aligners on 10M simulated HiSeq1000 Arabidopsis reads. (**D**) Percentage of the 10M simulated HiSeq1000 reads that were mapped correctly across various reads complexity level.

**Figure S6.** Amount of runtime conserved by post-processing optimization

**Supplementary Information:**

Availability and Requirements:

Project Name: BS Seeker3.

Project home page:

Operating system(s): Linux/Mac OS

Programming Language: Python 2.7+, C, gcc 5.4

Other requirements: pysam and matplotlib packages

License: MIT License

Any restrictions to use by non-academics: No

Testing Platform Specification: 80 Intel Xeon Cores, 180GB RAM

**Supplementary Note on Aligning to HG-19:**

In general, we are able to launch 2-3 sub-processes of BS-Seeker3 on the human genome on a ~180 GB machine without severely interfering with other activities. Roughly 80GB of memory would be required depending on specific parameters used to build the index. It should be noted that the decreased complexity actually plays well into the scheme, as the number of unique seeds is markedly reduced. BSseeker3 was designed to leverage the expanding RAM availability in the research community, and a high memory requirement for the human genome is the original sin that all aligners face.

**Supplementary Note on PBAT and RRBS Library:**

BS-seeker3 inherited all features from the previous version, BS-Seeker2. It can handle non-directional libraries but not PBAT libraries.

**Method 1.** BS-Seeker 3 Pipeline. (1) Improved Index Building: Two index instances instead of 4 are built based on the two three-letter converted genomes (C-to-T and G-to-A) based on both the Watson and Crick directions. (2) Fast Bisulfite Reads Mapping: The original raw reads file is split into smaller files based on available computational resources. Reads are transformed to the 3-letter code prior to fast mapping with SNAP. (3) Post-processing of SNAP Output: Each SNAP output file is further split and processed in parallel. We filter out reads that map to multiple locations or have too many mismatches in a highly optimized manner. (4) Display Methylation Statistics: We return a single-base-resolution report of the methylation level in this step. In BS Seeker3, at user’s discretion, we also estimate the un-conversion rate of the bisulfite treatment protocol, display a quality control plot of the reads, and output a metagene plot based on the methylation level for each site.

**Method 2.** Command Used to Run Each Benchmark Aligner. BS-Seeker 3 was benchmarked against 3 other aligners, BSMAP, Bismark, Brat-nova and BS-Seeker 2. For each aligner, their default settings were used and listed below.

BSMAP:

**./bsmap -a `BS-seq file` -d `reference genome fasta` -o `output tag` -n 0 -w 100 -v 5**

-n matching strand direction

-w maximum number of equal best hits to count

-v number of mismatches allowed per read

Bismark:

**./bismark --bowtie2 --sam -n 1 -l 32 `reference genome directory` `BS-seq file` --multicore 20**

--sam return output in sam format

-n the maximum number of mismatches allowed in the seed

-l the seed length

--multicore, number of used cores

BS-Seeker 2:

**python bs_seeker2-build.py –f `reference genome fasta` --aligner=bowtie2**

Brat-nova:

**./brat_bw -P `index directory` -s `file directory` -o `output name`**

BS-Seeker3:

**./bs3-align -i `file directory` -o WGBS -f `output format` -g `index directory`**

-o alignment with the whole-genome bisulfite sequencing mode

**Method 3.** Method Used to Generate Synthetic *Arabiodopsis* Reads. 10M simulated Illumina bisulfite reads from the *Arabiodopsis* library was generated using the Art software (Huang et all, 2012). We simulated complexity in the read data by varying the single base insertion/deletion rate in an average synthetic read. Three levels of data complexity (easy-to-align data, not-hard-to-align data, and difficult data) were simulated by three insertion/deletion rates (0, .001, .025). We simulated reads from both the HiSeq25 and HiSeq10 sequencers. Sample commands used by the Art software is given below:

**art_illumina -ss HS10 -sam –i genome.fa -l 100 -f 10 -ir 0.025 -dr .025 -o simulated025**

-ss HS10 was the sequencer simulated

-i genome.fa was the complete *Arabiodopsis* genome in fasta format

-l 100 was the length of each read

-f 10 was the read coverage

-ir was the single base pair insertion rate

-dr was the single base pair deletion

-o simulated025 was the synthetic read output

**art_illumina -ss HS25 -sam –i genome.fa -l 125 -f 20 -ir 0.001 -dr .001 -o simulated025**

-ss HiSeq2500 was the sequencer simulated

-i genome.fa was the complete *Arabiodopsis* genome in fasta format

-l 125 was the length of each read

-f 20 was the read coverage

-ir was the single base pair insertion rate

-dr was the single base pair deletion

-o simulated025 was the synthetic read output

**Method 4.** Method Used to Prepare Real Human Bisulfite Read Datasets. Real human bisulfite read datasets SRR019048.fastq , SRR018982.fastq, and SRR018985.fastq were downloaded from the European Bioinformatics Institute (Lister et all, 2009). SRR019048 contains 15,275,706 of 87bps reads, SRR018982.fastq contains 11,627,788, and SRR018985.fastq contains 11,972,696. The human genome is 3 billion base pairs long, so the ‘1X Human Genome’ data set contains approximately 35 million 87bps bisulfite reads, and the ‘.5X Human Genome contains approximately 17 million 87bps bisulfite reads. Each dataset was constructed by concatenating the downloaded real human read datasets.


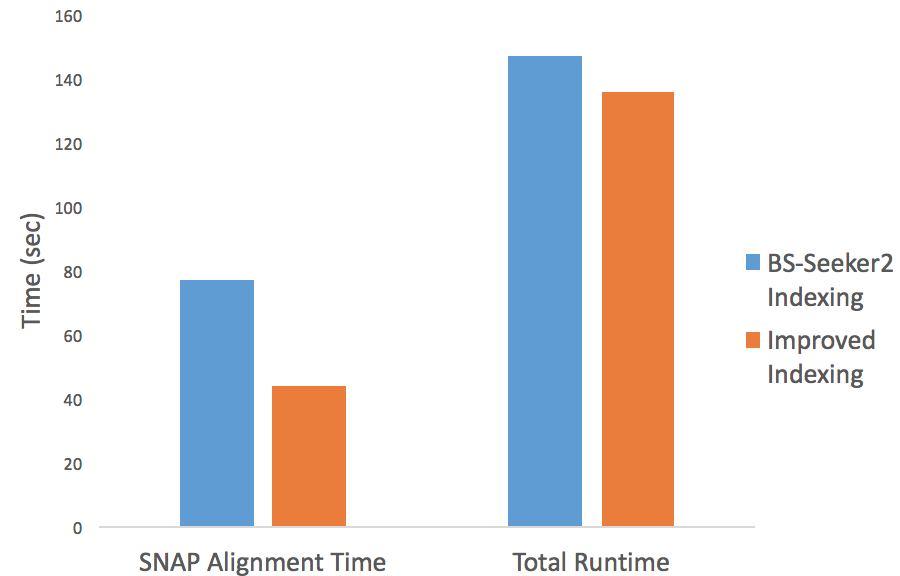


**147**

**137**

**77**

**47**

**Figure S1. Amount of runtime conserved by Improved Indexing.** With the improved indexing, the time spent on raw-read alignment was conserved by 44%. The total runtime is conserved by 7%. We tested the effect of improved indexing with the 10M synthetic *Arabiodopsis* data generated by Supplementary Method 1.

B

A


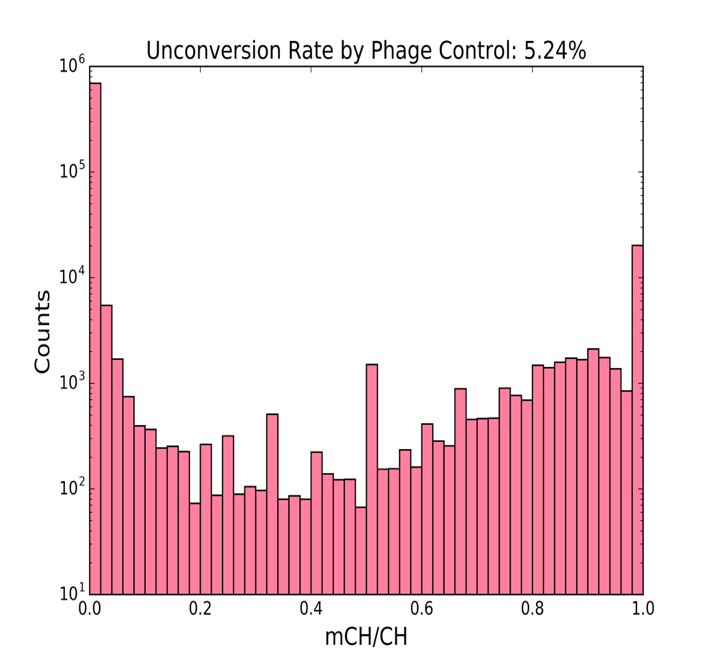

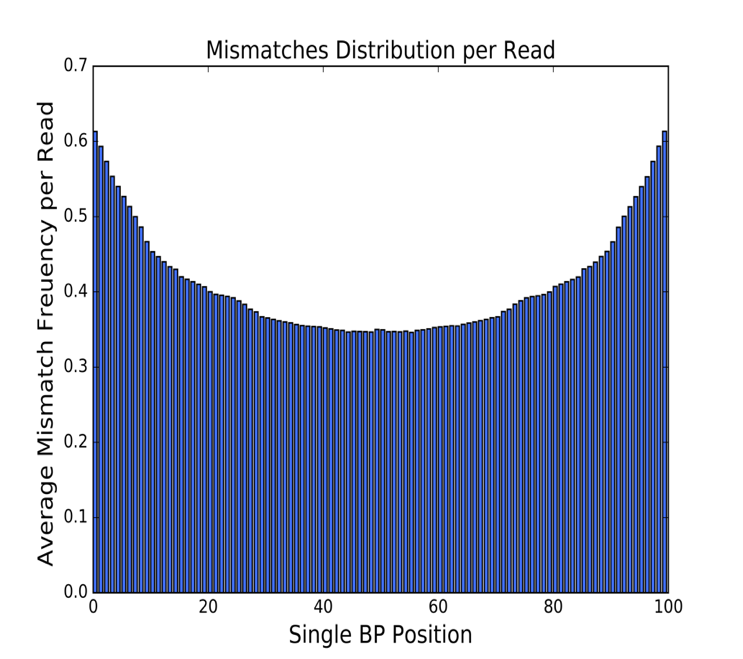


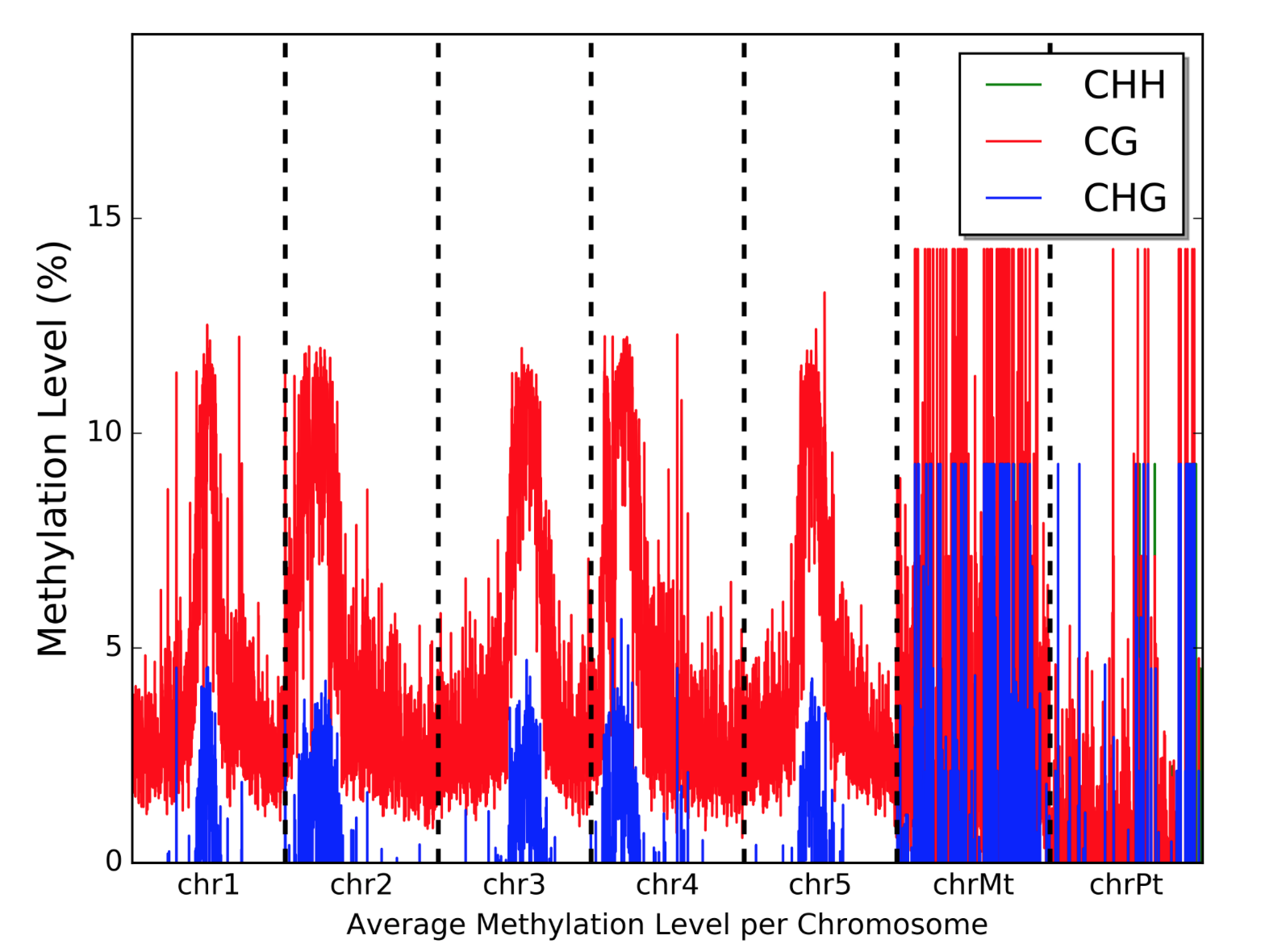


C

**Figure S2 Additional analysis of BS-seq Data** A) Un-conversion Rate Plot: This plots shows the distribution of the unconverted ratio of CH sites (H = A, C, T) in the input reads. The input reads (containing reads form the lamda phage library) are mapped against phage reference genome, which has been found to be free of DNA methylation. B) QC Plot: This plot displays the cumulated number of mismatches along each read position for all uniquely mapped reads outputted by SNAP. The read length is supplied by the user. C) Average Chromosomal View of the Methylation Level Distribution: The methylation level distribution along each 1000 bps interval of the genome is averaged and displayed if the user did not specify a genomic structure and supply a gene annotation file.


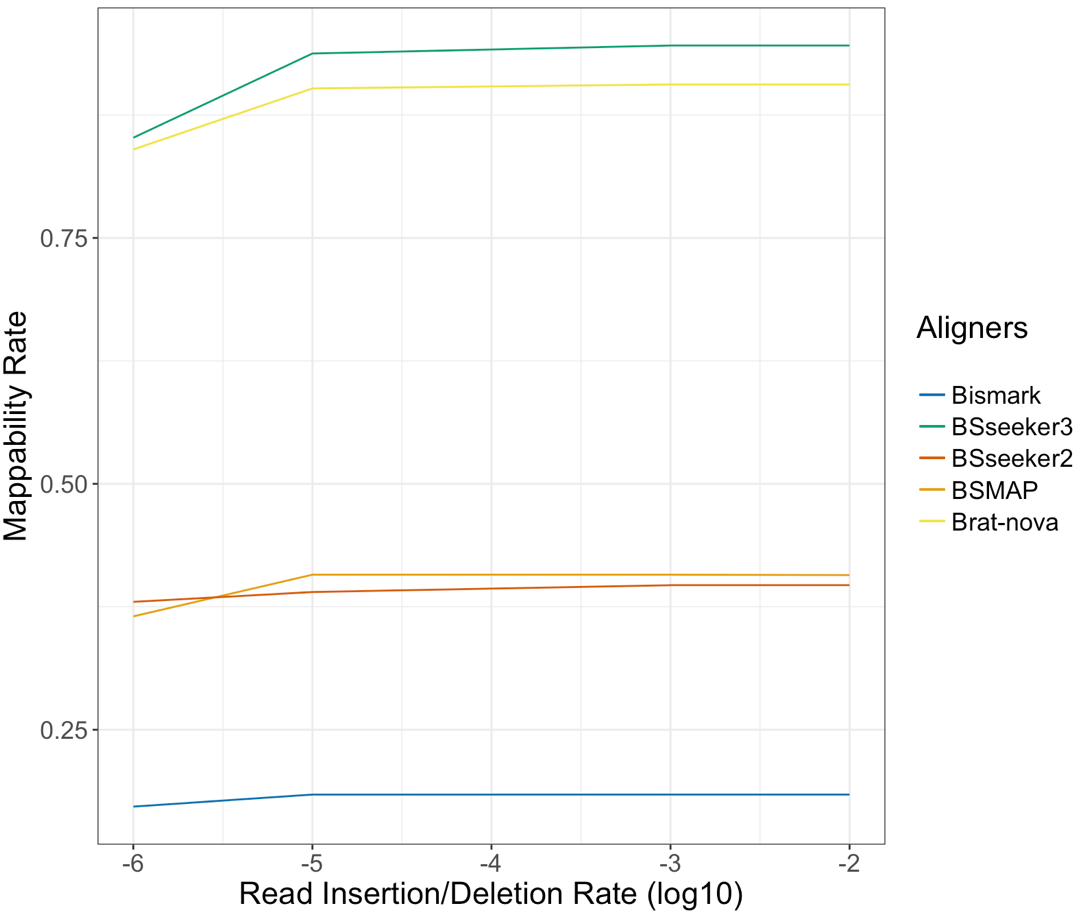


**Figure S3.** Mapibility rate of the 10M simulated HiSeq1000 reads by BSseeker3, BSseeker2, BSMAP, Brat-Nova, and BSMAP across different data complexity level. BS-Seeker3 and Brat-nova produced the highest mappability rate on a consistent basis across all dataset. BS-Seeker2 and BSMAP had similar mappability rate while Bismark mapped the least number of reads.


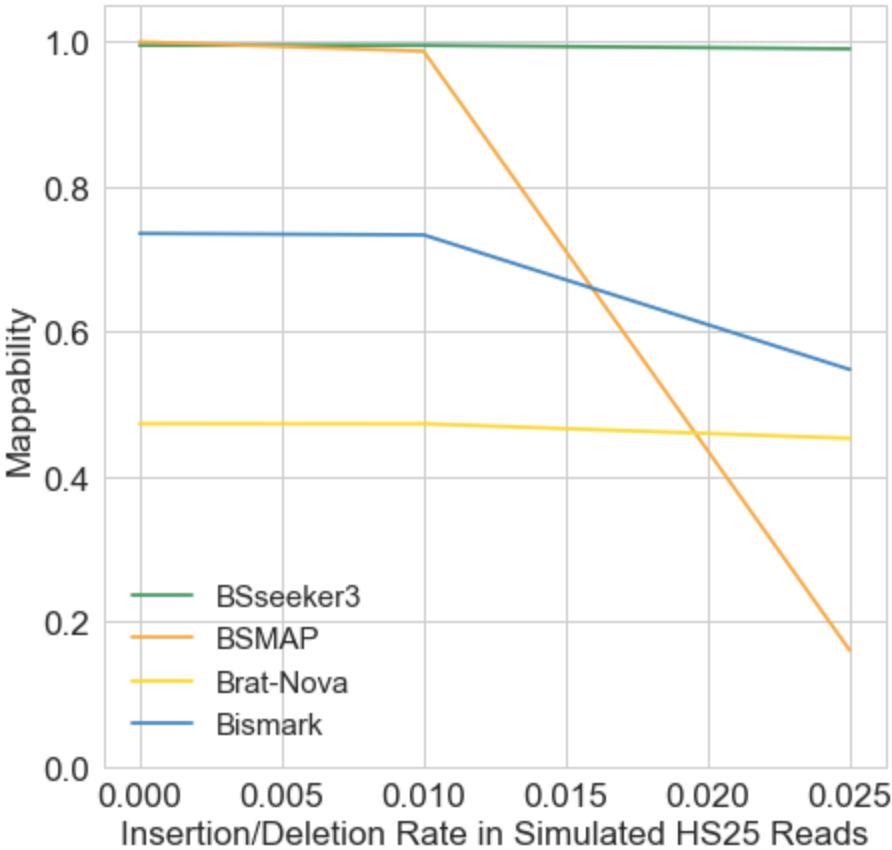


**Figure S4.** Mapibility rate of the 10M simulated HiSeq25 reads by BSseeker3, BSMAP, Brat-Nova, and BSMAP across different data complexity level. BSMAP and BSseeker3 mapped the most reads at low level of indel rate (0, .005) in the reads. After that, BSMAP’s mapping rate sharply decreases, while BS-Seeker3 continued to map efficiently at 99%. Note-worthily, Brat-Nova performed worse than with HiSeq1000 data, but maintained a constant mapping rate above .4 even at high indel rates.

**
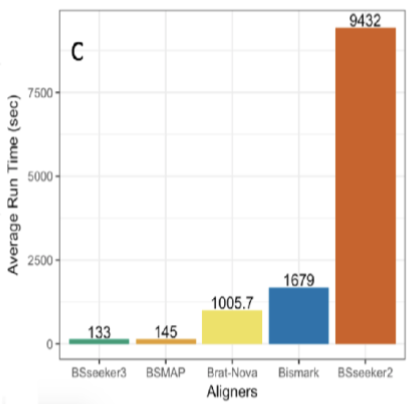

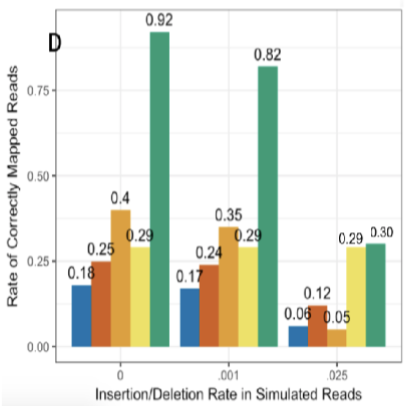
**

**Figure S5**. (**C**) Average user runtime of the four aligners on 10M simulated HiSeq1000 Arabidopsis reads. (**D**) Percentage of the 10M simulated HiSeq1000 reads that were mapped correctly across various reads complexity level.

**Figure S6.** Amount of runtime conserved by post-processing optimization. With the post-processing optimization, the total alignment runtime was reduced by a factor of ¼. We tested the effect of post-processing with the 12M synthetic reads generated by Supplementary Method 1.
